# Comparative Screening of the Liver Gene Expression Profiles from Type 1 and Type 2 Diabetes Rat Models

**DOI:** 10.3390/ijms25084151

**Published:** 2024-04-09

**Authors:** Paloma Lucía Guerra-Ávila, Tereso J. Guzmán, Belinda Vargas-Guerrero, José Alfredo Domínguez-Rosales, Alejandra Beatriz Cervantes-Garduño, Adriana María Salazar-Montes, Laura Verónica Sánchez-Orozco, Carmen Magdalena Gurrola-Díaz

**Affiliations:** 1 Instituto de Investigación en Enfermedades Crónico-Degenerativas, Instituto Transdisciplinar de Investigación e Innovación en Salud, Departamento de Biología Molecular y Genómica, Centro Universitario de Ciencias de la Salud (C.U.C.S.), Universidad de Guadalajara, Guadalajara, Sierra Mojada 950, Puerta peatonal 7, Col. Independencia, Guadalajara C.P. 44350, Mexico; paloma.guerra3434@alumnos.udg.mx (P.L.G.-Á.); goozman57@hotmail.com (T.J.G.); belinda.vargas@academicos.udg.mx (B.V.-G.); jose.drosales@academicos.udg.mx (J.A.D.-R.); adriana.smontes@academicos.udg.mx (A.M.S.-M.); laura.sorozco@academicos.udg.mx (L.V.S.-O.); 2 Department of Pharmaceutical and Medicinal Chemistry, University of Münster, Corrensstraße 48, 48149 Münster, Germany; 3 Laboratorio de Genómica Clínica, Facultad de Odontología, Universidad Nacional Autónoma de México, Coyoacán, Ciudad de México C.P. 04510, Mexico; alejandra.cervantes@fo.odonto.unam.mx

**Keywords:** high-fat diet (HFD), insulin resistance (IR), streptozotocin (STZ), type 2 diabetes (T2D), type 1 diabetes (T1D), Wistar rats

## Abstract

Experimental animal models of diabetes can be useful for identifying novel targets related to disease, for understanding its physiopathology, and for evaluating emerging antidiabetic treatments. This study aimed to characterize two rat diabetes models: HFD + STZ, a high-fat diet (60% fat) combined with streptozotocin administration (STZ, 35 mg/kg BW), and a model with a single STZ dose (65 mg/kg BW) in comparison with healthy rats. HFD + STZ- induced animals demonstrated a stable hyperglycemia range (350–450 mg/dL), whereas in the STZ-induced rats, we found glucose concentration values with a greater dispersion, ranging from 270 to 510 mg/dL. Moreover, in the HFD + STZ group, the AUC value of the insulin tolerance test (ITT) was found to be remarkably augmented by 6.2-fold higher than in healthy animals (33,687.0 ± 1705.7 mg/dL/min vs. 5469.0 ± 267.6, respectively), indicating insulin resistance (IR). In contrast, a more moderate AUC value was observed in the STZ group (19,059.0 ± 3037.4 mg/dL/min) resulting in a value 2.5-fold higher than the average exhibited by the control group. After microarray experiments on liver tissue from all animals, we analyzed genes exhibiting a fold change value in gene expression <−2 or >2 (*p*-value <0.05). We found 27,686 differentially expressed genes (DEG), identified the top 10 DEGs and detected 849 coding genes that exhibited opposite expression patterns between both diabetes models (491 upregulated genes in the STZ model and 358 upregulated genes in HFD + STZ animals). Finally, we performed an enrichment analysis of the 849 selected genes. Whereas in the STZ model we found cellular pathways related to lipid biosynthesis and metabolism, in the HFD + STZ model we identified pathways related to immunometabolism. Some phenotypic differences observed in the models could be explained by transcriptomic results; however, further studies are needed to corroborate these findings. Our data confirm that the STZ and the HFD + STZ models are reliable experimental models for human T1D and T2D, respectively. These results also provide insight into alterations in the expression of specific liver genes and could be utilized in future studies focusing on diabetes complications associated with impaired liver function.

## 1. Introduction

Diabetes is a complex metabolic disorder characterized by chronic and irreversible hyperglycemia. In type 1 diabetes (T1D), pancreatic β-cell damage and cell death are primarily mediated by immune processes, resulting in a significant loss of β cells. In contrast, type 2 diabetes (T2D) involves the dysfunction and a partial reduction in β cells mass, as well as metabolic factors that lead to insulin resistance. At the cellular level, endoplasmic reticulum stress impacts β cells in both diabetes types. In T1D, β-cell damage is predominantly mediated by the IRE1 pathway, while in T2D, the PERK-eIF2α pathway primarily mediates β-cell damage [1]. With respect to the liver, T1D is associated with abnormal hepatic glycogen metabolism. Insufficient insulin and excessive glucagon promote hepatic lipolysis and ketogenesis, thereby reducing glycogen synthesis. On the other hand, T2D is associated with ectopic lipid accumulation, insulin resistance, and consequently reduced glycogen synthesis. Particularly in T2D, increased hepatic lipogenesis and liver steatosis lead to reduced insulin clearance and sensitivity, a phenomenon not observed in T1D [2]. These data highlight that although there are some cellular pathways in common for both diabetes types, it is important to understand the impact of the differential gene expression between T1D and T2D.

The implementation of diabetes animal models is essential to identify and understand cellular and molecular mechanisms involved in the development and progression of this disease. The use of these models also allows the investigation of emerging antidiabetic compounds that could complement the conventional clinical management of this pathology [3,4]. The most used chemical compound in experimental diabetes induction is streptozotocin (STZ), which exerts specific toxicity in pancreatic β cells. STZ is obtained from the fungus *Streptomyces achromogenes* and is internalized into the pancreatic β cells by the glucotransporter 2 (GLUT2), inducing DNA alkylation and apoptosis [5]. However, it has been observed that at high doses (>65 mg/kg BW), STZ frequently leads to a phenotype resembling type 1 diabetes (T1D). In contrast, STZ at low doses (<35 mg/kg BW) has been reported to decrease insulin secretion and could resemble a late stage of type 2 diabetes (T2D). Regardless of the STZ dose, this compound alone does not lead to the development of insulin resistance (IR), a typical T2D characteristic [6]. On the other hand, some animal models have been established with the inclusion of special diets such as the high-fat diet (HFD) or high-carbohydrate diet (HCHD) before chemical diabetes induction (STZ). In general, HCHD models resemble metabolic syndrome, a condition that precedes T2D. In contrast, HFD + STZ models in rats can closely reproduce human T2D features [7]. In terms of fat content, diets can be classified as a low-fat diet (LFD, 10–30%), high-fat diet (HFD, 30–50%), or very high-fat diet (VHFD, >50%). In contrast, a standard chow diet contains up to 10% fat. It is widely accepted that HFD and VHFD are the most suitable diets for inducing T2D. Moreover, the influence of HFD on the metabolism depends on the type of fat included in the diet. Dietary fat derived from polyunsaturated ω-3 fatty acids from vegetable oils, such as coconut oil or fish oil, may even exert beneficial effects on body composition and insulin sensitivity [8]. Meanwhile, animal fat, such as lard (composed of equal proportions of saturated and monounsaturated fatty acids), can lead to obesity and the development of insulin resistance [9].

Here, we compared blood glucose homeostasis, biochemical, histopathological, and immunohistochemical parameters in the two diabetes models. We demonstrate that the HFD + STZ model is more consistent in the in vivo development of hyperglycemia and exhibits concomitant insulin resistance, in comparison to the STZ model. This indicates that the HFD + STZ model manifests similar characteristics to those found in human T2D patients, with an impairment in metabolic homeostasis that involves T2D complications such non-alcoholic fatty liver disease (NAFLD), dyslipidemia, and obesity. Furthermore, our study was focused on the analysis of liver tissue since this organ plays an important role in the homeostasis of carbohydrates and lipids and its function impairment correlates with the development of metabolic disorders such as T2D [10]. In fact, NAFLD has been associated with macro- and microvascular diabetes complications, such as cardiovascular and chronic kidney diseases [11]. Ectopic lipid accumulation in the liver promotes NAFLD involving insulin resistance and chronic low-grade inflammation [12]. Likewise, adipose tissue dysfunction is closely related to NAFLD, enhancing pro-inflammatory, diabetogenic, and atherogenic responses [13,14].

On the other hand, the observed phenotypic characteristics from diabetic animals are a result of molecular modifications, such as a different gene expression profile that alters the metabolic homeostasis. In this regard, microarray analysis offers a useful platform to explore the gene expression pattern in experimental diabetic rats compared with healthy animals. To perform a non-biased analysis and comparison of high throughput gene expression data collected from T1D and T2D experimental models, it is imperative to employ a similar analytical platform. Previously, the gene expression profile of animals induced to diabetes by STZ was reported [15]. Similarly, a transcriptome analysis on the liver of HFD + STZ rats was performed previously [16]. However, our study provides an unbiased comparison of the gene expression modulation promoted in both induction models. We employed a single microarray format, allowing us to minimize potential errors and increase the validity of comparable results. In addition, the genes identified and employed for the enrichment analysis provide a novel insight into the main molecular and cellular differences in the gene signature of both models. Consequently, we screened differences in liver gene expression in experimental STZ and HFD + STZ diabetic models.

## 2. Results

### 2.1. STZ Administration Reduced the Body Weight Gain in the STZ Model

**Figure 1:**
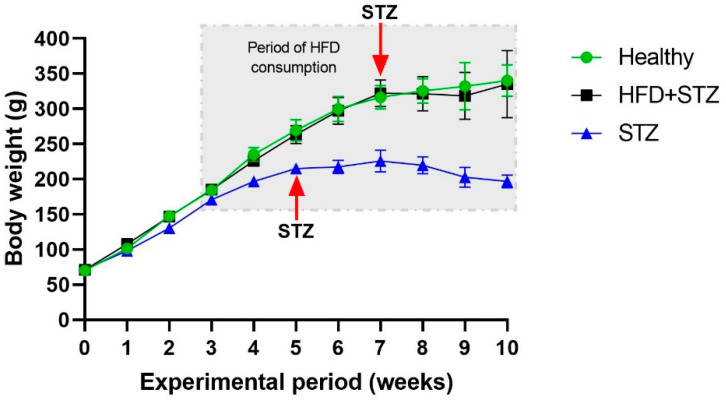
Comparison of body weight gain among the experimental groups. n = 9 animals per experimental group.

Body weight increased gradually in both models, although in HFD + STZ animals, the body weight was higher than in STZ animals (Figure 1). After STZ administration in the STZ model, a negative impact on the body weight gain was observed.

### 2.2. Hyperglycemia Is Maintained within a Narrower Value Range in the HFD + STZ Model Compared to the STZ Model

**Figure 2:**
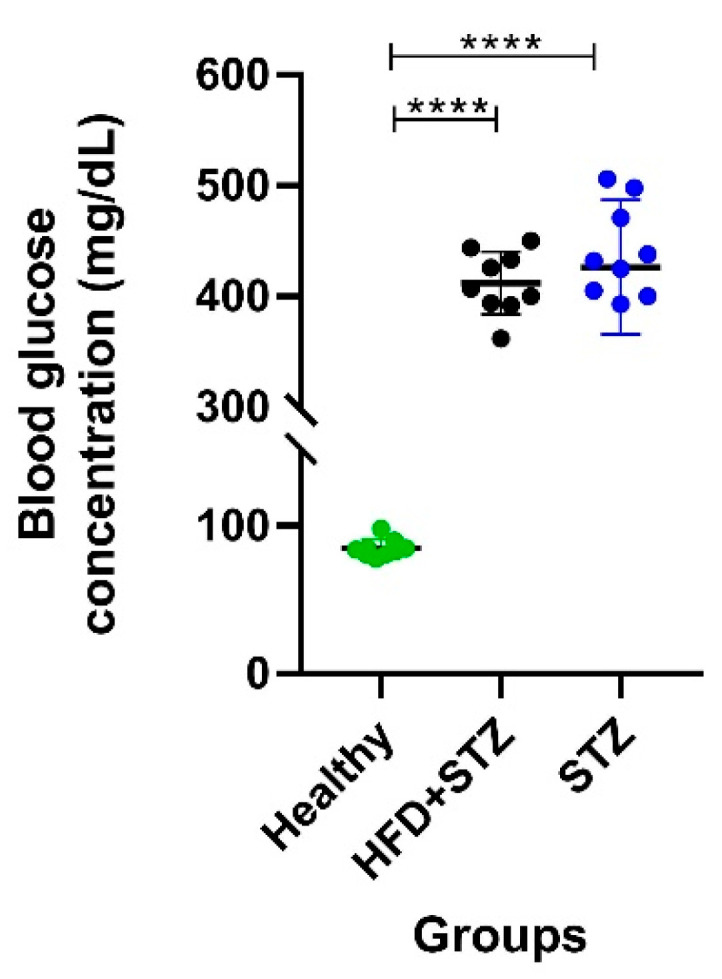
Comparison of the distribution of glycemia values in all experimental groups, one week after a single dose of STZ. STZ: diabetes induction with streptozotocin (65 mg/kg BW). HFD + STZ: induction with a combination of a high-fat diet (60% fat) + streptozotocin (35 mg/kg BW), n = 9 animals per experimental group. ANOVA test and Tukey’s post hoc test, **** *p* < 0.0001.

As shown in Figure 2, the HFD + STZ model revealed homogeneous post-induction blood glucose levels (within a range of 350 to 450 mg/dL, and a mean of 412.0 ± 9.5 mg/dL) and a maximum difference of 100 mg/dL between the lowest and the highest blood glucose concentration. On the other hand, in the STZ model, we found glucose concentration values with a greater dispersion, ranging from 270 to 510 mg/dL, with a mean of 426.3 ± 19.2 mg/dL. The latest results exhibit a wide range of glucose concentration values, approximately 250 mg/dL, representing a glucose difference of two and a half times higher than that found in the HFD + STZ model (Figure 2).

### 2.3. HFD + STZ and STZ Models Exhibit Impaired Glucose Tolerance but Insulin Resistance Is Only Found in the HDF + STZ Animals

**Figure 3:**
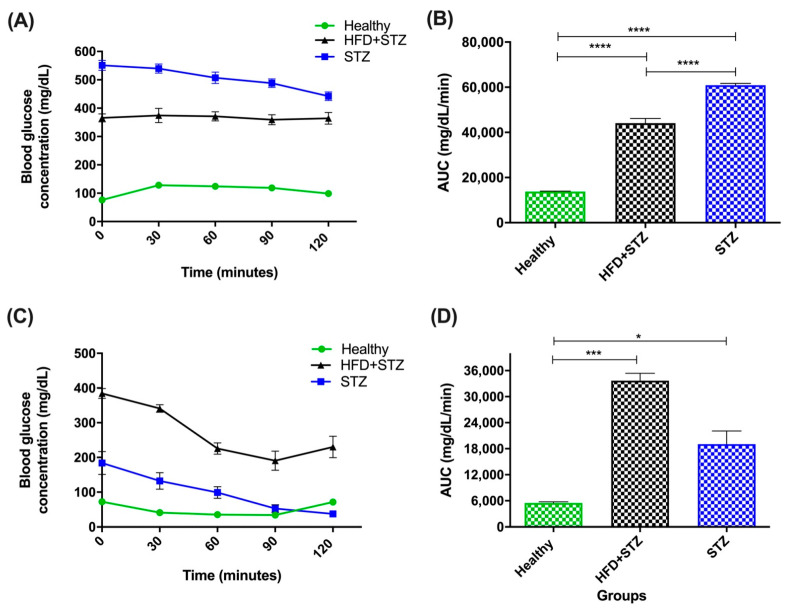
Glucose homeostasis assessment of all experimental groups. (**A**) Oral glucose tolerance test (OGTT), (**B**) area under the curve of OGTT, (**C**) insulin tolerance test (ITT), and (**D**) area under the curve of ITT (mean ± SEM). HFD + STZ: high-fat diet plus streptozotocin group, STZ: streptozotocin group, n = 9 animals per experimental group. * *p* < 0.05, *** *p* < 0.001, **** *p* < 0.0001.

In the HFD + STZ model, we observed that blood glucose concentration remained within a stable hyperglycemia during the 120 min of the test. In contrast, in the STZ model, gradually decreasing glucose levels were found. Despite these differences in both models, HFD + STZ and STZ rats showed impaired glucose tolerance in comparison to healthy animals (Figure 3A).

In Figure 3B, area under the curve (AUC) values from the OGTT data (Figure 3A) were calculated. As expected, AUC values were significantly higher in STZ rats (60,933.0 ± 794.7 mg/dL/min) than in HFD + STZ animals (44,100.0 ± 2085.7 mg/dL/min). This can be explained by the higher glucose values exhibited by the STZ rats, compared to HFD + STZ animals.

To assess the extent of insulin resistance in the experimental models, we performed the insulin tolerance test after i.p. insulin administration. In this test, HFD + STZ rats responded with continuous hyperglycemia during the evaluated period (120 min); however, animals did not reach normoglycemic values (<100 mg/dL). It should be noted that the glucose levels remain above 200 mg/dL (experimental T2D cut-off value) in HFD + STZ animals. On the contrary, the blood glucose levels of STZ animals decreased rapidly, even reaching hypoglycemic-like glucose values. This difference could be explained by the severity of β-cell damage in the STZ model and the poor insulin response in the peripheral organs of HFD + STZ diabetes-induced rats (Figure 3C).

In the HFD + STZ group, the AUC value of the ITT is remarkably augmented by 5.2-fold higher than in healthy animals (33,687.0 ± 1705.7 mg/dL/min vs. 5469.0 ± 267.6 respectively), indicating insulin resistance (IR). In contrast, a moderated AUC value was observed in the STZ group (19,059.0 ± 3037.4 mg/dL/min) resulting in 2.5-fold more than the average exhibited by the control group (Figure 3D).

### 2.4. Comparison of Biochemical Parameters in the Experimental Diabetes Models

Blood biochemical parameters were quantified pre- and post- induction in all experimental groups, as shown in Table 1. As expected, STZ administration induced hyperglycemia in both models, but this was exacerbated in HFD + STZ animals. Serum glucose levels were increased by 52.4% in the STZ rats, whereas 66.5% was observed in the HFD + STZ animals. In fact, both models exhibited an adequate diabetes induction considering the established cut-off value to confirm diabetes in rats (>200 mg/dL) [17]. Regarding the serum lipid profile, HFD + STZ rats showed an increase in total cholesterol concentration and consequently an increase also in LDL-c levels. The concentration of these analytes remained unchanged in the STZ model. Notably, triglycerides increased in both rat models, but this percentage was higher in the HFD + STZ model (101.2% vs. 35.2%). Putative renal damage was found only in the STZ model, as indicated by increased urea levels.

### 2.5. Hepatomegaly and Liver Steatosis Were Only Developed in the HFD + STZ Model

**Figure 4:**
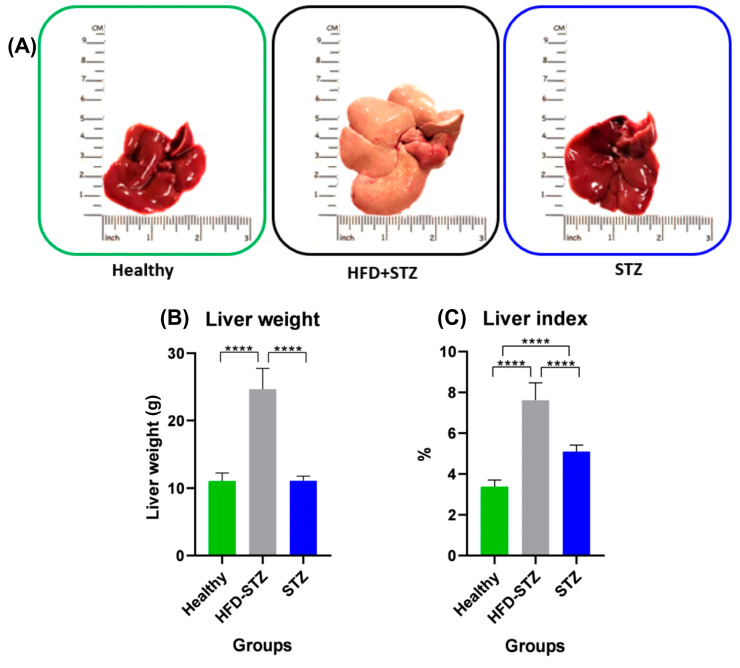
Analysis of the macroscopic characteristics of the liver in all experimental groups. (**A**) Representative images of the liver from all experimental groups. (**B**) Liver weight (g) and (**C**) liver index (%) of the healthy group as well as the HFD + STZ and STZ groups. HFD + STZ: high-fat diet plus streptozotocin, STZ: streptozotocin, n = 9 animals per experimental group. **** *p* < 0.0001.

At the end of the experimental period, livers from HFD + STZ rats exhibited a macroscopic appearance of hepatic steatosis (Figure 4A, middle panel). On the contrary, the normal macroscopic characteristics of the liver were similar in healthy and STZ animals (Figure 4A). Moreover, hepatic organs were weighed, and their mean weights were similar in healthy animals and STZ rats (10.7 ± 0.4 g and 11.1 ± 0.2 g, respectively). In contrast, the liver from HFD + STZ rats reached a weight of 25.0 ± 1.0 g and a marked increase in the organ size, suggesting hepatomegaly (Figure 4B). As shown in Figure 4B, the liver weight of healthy and STZ rats did not show any increase. On the contrary, the liver index was modified since STZ rats lost body weight during the experimental induction (Figure 4C) and a discrete increase in the liver index of 5.1 ± 0.1% was found in STZ rats. Moreover, the liver index of the HFD + STZ animals exhibited an increase in this value (7.6 ± 0.3%).

### 2.6. Liver Steatosis Is Clearly Appreciated in the HFD + STZ Animals

**Figure 5:**
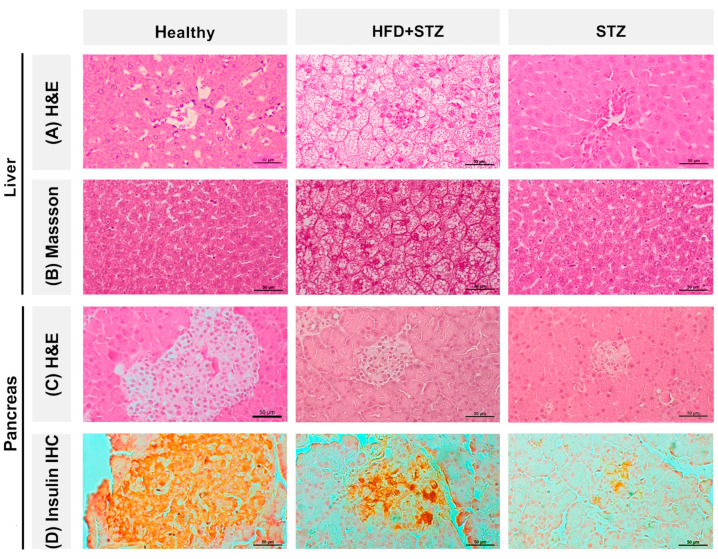
Histopathological changes in hepatic and pancreatic tissues from all experimental groups. Representative photomicrographs of liver tissue stained with (**A**) hematoxylin and eosin (H&E, 40×) and (**B**) Masson’s trichome (40×). Representative photomicrographs of pancreatic tissue stained with (**C**) hematoxylin and eosin (H&E, 40×) and (**D**) insulin immunoreactive pancreatic islet cells (40×): Healthy group, 80% insulin-positive cells; HFD + STZ group, 40% insulin-positive cells; and STZ group, 15% insulin-positive cells (data represent the mean of the immunopositivity percentage).

After histological analysis, we found that the tissue architecture of livers from HFD + STZ animals was markedly altered compared to the healthy group and the STZ model. After the HFD + STZ induction, we observed steatosis in the panacinar liver tissue, as well as its compatible characteristic such as lobular inflammation and prominent cell ballooning (Figure 5A). In contrast, the hepatic tissue from STZ animals displayed a normal histological architecture consisting of hepatic lobules with a normal central vein. Concerning Masson’s trichrome staining, we did not find any relevant collagen deposits or other indicators of fibrosis in the analyzed tissues (Figure 5B). 

### 2.7. The Pancreas of STZ Rats Contains a Reduced Number of Insulin-Positive Cells Compared to That of the HFD + STZ Model

Firstly, Figure 5C shows the H&E-stained slides of the pancreas where islets of Langerhans are observed in the different animal groups. The islets of Langerhans of healthy rats are generally larger with round endocrine cells with a central nucleus, homogeneous chromatin of eosinophilic cytoplasm in cells in the center of the islet, and clear cytoplasm in the periphery with capillaries located between the endocrine cells. Rats of the HFD + STZ group had smaller, fewer endocrine cells in their islets of Langerhans, and their cellular morphology was altered. These changes included nuclear pleomorphism, heterogeneous nuclear chromatin, the presence of lymphocytes, and a lack of capillaries. Remarkably, STZ rats have smaller pancreatic islets and cellular alterations, like those of rats of the HFD + STZ group but to a greater extent.

Insulin immunohistochemistry (IHC) was performed to assess the number of insulin-positive cells, their insulin immunostaining-intensity, and their cellular localization in the pancreatic tissue.

We found that the number of insulin-positive cells and their intensity were significantly reduced in the pancreas of HFD + STZ and STZ rats compared to the healthy group. These findings are consistent with the morphological changes observed in the HE-stained sections from pancreatic tissues of diabetic animals. After IHC analysis, a value of 80%, in terms of the proportion of insulin-positive cells, was observed in the pancreatic islets from animals of the healthy group. By contrast, tissues from HFD + STZ animals showed a reduction of 40%. This reduction was more drastic in tissues from STZ rats, resulting in 15% of insulin-positive cells (Figure 5D).

### 2.8. Liver Gene Expression Profile by DNA Microarrays

After evaluation of the biochemical, metabolic, and histological parameters across all animal groups, we explored the alterations in the gene expression profile post-diabetes induction, in comparison to healthy animals. The gene expression profile was scrutinized in the three experimental groups: (1) Healthy animals; (2) HFD + STZ animals (resembling type 2 diabetes); and (3) STZ animals (simulating type 1 diabetes). Initially, we evaluated the integrity of RNA (RIN) of each sample. All RNA samples exhibited an RNA integrity number (RIN) > 8.8, indicating an optimal RNA quality (Figures S1 and S2). Individual samples from each group were pooled and subsequently analyzed by DNA microarrays (Clariom D array, rat). For analysis of gene expression data, we used Affymetrix software (Transcriptome Analysis Console, TAC, v.4.0.2, ThermoFisher Scientific, Santa Clara, CA, USA). Condition pairings revealed differentially expressed genes (DEGs) and the top 10 up- and downregulated genes for each experimental group. Subsequently, TAC, v.4.0.2, generated graphs of sample signals for comparison across all experimental groups. We identified the genes that altered their expression after diabetes induction as well as the coding genes that were inversely expressed in HFD + STZ and STZ groups. Lastly, we focused on the functional enrichment analysis conducted in STRING to identify metabolic pathways implicated in diabetes development in every diabetes model.

Transcriptome Analysis Console v. 4.0.2

**Figure 6:**
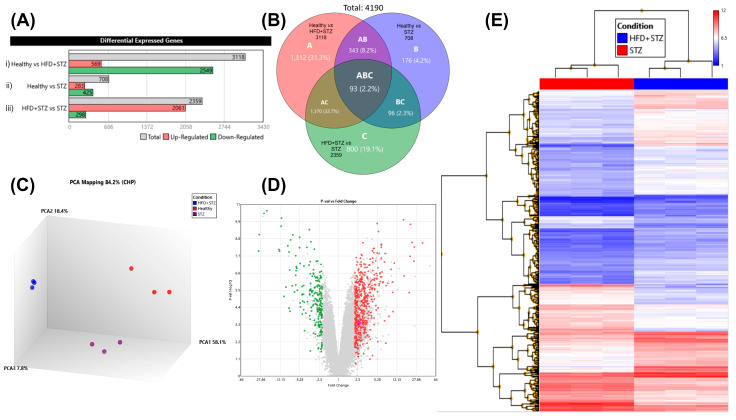
Primary analysis provided by TAC. A summary of the genes that were upregulated and downregulated in the experimental groups. (**A**) Differentially expressed genes (DEGs) after microarray analysis among healthy, HFD + STZ, and STZ groups, n = 9 animals per experimental group. (**B**) Venn diagram depicting the number of DEGs among the experimental groups. (**C**) Distribution of three biological replicates from each group evaluated by principal component analysis (PCA). (**D**) Volcano plot including 68,842 genes and showing the up- (red) and down- (green) regulated coding genes (**E**) Hierarchical clustering displayed homogeneity and reproducibility of the gene expression pattern from triplicates in HFD + STZ (blue) and STZ (red) conditions.

Gene expression data were analyzed using the transcriptome analysis console (TAC, v.4.0.2), yielding a total signal from 68,842 genes. TAC, v.4.0.2 software was used to analyze and obtain results and graphics shown in this section (Figure 6). Firstly, we sought targets related to diabetes within the range of a classic fold change (>2 or <−2, *p* < 0.05). After that, 27,686 differentially expressed genes (DEGs) were found. DEGs were evaluated in pairs to explain how the HFD + STZ and STZ models differed from the hepatic gene expression profile of healthy animals (3118 and 708 DEGs, respectively, Figure 6A(i,ii). Figure 6A(iii) shows the comparison of both models: HFD + STZ versus STZ. Following this last comparison, the remaining gene expression data were: 2359 DEGs (representing 3.43% of the total genes, or 2359/68,842). Of these, 2061 genes (87.4%) were upregulated, and 298 genes (12.6%) were downregulated (Figure 6A(iii)). A Venn diagram, displayed by TAC, illustrates the total number of DEGs in each experimental group (Figure 6B). Principal component analysis (PCA) was employed to summarize the large and heterogeneous microarray datasets, reducing their dimensionality for easier interpretation. Once the dimensionality was reduced, each sample was assigned to a group or cluster. The PCA of our results highlights the overall difference in gene expression profiles between groups (Figure 6C). After applying a filter that selects only coding genes from the total of genes (68,842), a volcano plot was generated that revealed 498 upregulated (red) and 213 downregulated (green) coding genes, which differ in both models (Figure 6D). Hierarchical clustering was conducted to illustrate the variations in gene expression patterns between experimental groups. We examined the gene expression patterns in two diabetes-induced groups, namely the HFD + STZ group (represented in blue) and the STZ group (depicted in red). Each condition was analyzed by triplicates, with each triplicate considered as an independent sample. The reproducibility and homogeneity of the gene expression pattern of each independent triplicate, in each condition, are clearly depicted in Figure 6E.

After applying a filter that selects only coding genes, the differentially regulated genes that exhibited the highest significant fold change values were identified. Firstly, the comparative analysis revealed that the pair: healthy vs. HFD + STZ yielded 1039 coding genes where it was a reduction in the number of genes (from 3118, (Figure 6A(i)) to 1039). Consequently, pair healthy vs. STZ yielded 242 genes (from 708, (Figure 6A(ii)), whereas pair HFD + STZ vs. STZ, 706 genes (from 2359, (Figure 6A(iii)).

Table 2 presents the 10 coding genes that were the highest up- and downregulated when the experimental groups were analyzed in pairs. 

Diabetes, when it is experimentally induced in laboratory animals, can resemble either type 1 or type 2 diabetes (T1D or T2D). In the chemical induction of diabetes, these phenotypes strongly depend on factors such as the induction substance and its dose, among others. In addition, the diet can play an important role in the development of diabetes. As a result, we aimed to identify characteristic gene clusters that could differentiate between T1D and T2D models. For this analysis, we evaluated a total of 24,753 coding genes. To screen the gene expression profile, we compared the individual sample signals (expression levels) of each gene, generated in a TAC. The sample signal chart displays the expression levels of one individual gene in each group (Figures S3 and S4). We identified genes that altered their expression after diabetes induction, compared to healthy animals, which were used as a reference for gene expression. Of particular interest was the analysis of genes that exhibited opposite expression levels between both experimental diabetes models. This means that one gene can be overexpressed in the HFD + STZ model, but to be considered in this analysis, it needs to be low expressed in the STZ model and vice versa. A total of 849 genes exhibited opposite expression patterns between both models. From these genes, we found 358 upregulated genes in the HFD + STZ model (which were downregulated in STZ), and 491 upregulated genes in the STZ model (which were downregulated in HFD + STZ). A complete list of these genes is provided in the Supplementary Materials (Table S1).

Later, two enrichment analyses were conducted using the STRING database. The first analysis assessed genes that were upregulated in T1D (STZ, 491 genes) and downregulated in T2D (HFD + STZ, 358 genes), Table 3. Conversely, in a second analysis, we evaluated the genes that were upregulated in T2D (HFD + STZ, 358 genes) and downregulated in T1D (STZ, 491 genes), Table 4. Subsequently, functional enrichment analyses were performed on both sets of genes, using gene ontology (GO), mammalian phenotype ontology, the Kyoto Encyclopedia of Genes and Genomes (KEGG), and WikiPathways.

Additionally, a comprehensive list of genes exhibiting similar up- and downregulated expression patterns in HFD + STZ and STZ animals is provided in Tables S2 and S3.

## 3. Discussion

In this experiment, we compared blood glucose homeostasis and biochemical, histopathological, and immunohistochemical parameters in two experimental diabetes models. We found the HFD + STZ model to be more consistent with respect to the development of hyperglycemia and to exhibit concomitant insulin resistance. In addition, to obtain a more comprehensive understanding of the molecular mechanisms involved in the diabetic phenotype, we performed an analysis of the liver gene expression profile in the tissues obtained from diabetic animals. Among the existing experimental diabetes models, the combination of a high-fat diet with a low dose of STZ administration has been suggested as a suitable type 2 diabetes (T2D) model [6]. However, there are several methodological differences described for the induction of this model. According to the literature, we found that the duration of the diet period before STZ administration is an important factor for optimal diabetes induction. Likewise, we observed that in the establishment of a stable diabetes animal model, other factors, such as the percentage and source of fat, the inclusion of cholesterol and sodium cholate, diet elaboration, and the time of consumption before STZ administration, are also relevant. Moreover, our findings are in accordance with previous studies, suggesting that a 4-week period of HFD consumption prior to STZ administration is sufficient to develop IR [8,18,19,20,21,22,23,24,25,26]. In our study, we formulated the HFD with lard as the fat source, composed of equal proportions of saturated and monounsaturated fatty acids (FAs). These types of fatty acids lead to the most pronounced manifestations of obesity, IR, and metabolic syndrome [8]. Indeed, livers from the HFD + STZ rats, but not from the STZ rats, exhibited a macroscopic appearance of hepatic steatosis. Similar macroscopic findings were reported in livers from rats that were fed only with an HFD, exhibiting increased fat deposition characterized by macroscopic white dots, an enlarged mass, and a paler and softer hepatic appearance [27,28,29,30,31]. In addition, the liver index evaluation of our results agrees with those reported in previous studies [30,32,33].

OGTT results in our HFD + STZ rats are in agreement with studies examining blood glucose levels in similar HFD + STZ models, indicating a glucose metabolism impairment [33,34,35,36,37,38]. These comparisons support the impaired glucose tolerance exhibited by both experimental diabetes models. Regarding biochemical parameters, we observed similar total cholesterol, LDL-c, and triglyceride levels in comparable HFD + STZ rat models [39,40,41]. At the level of histopathology, the disarrayed hepatic architecture in the HFD + STZ rats’ tissue is consistent with that reported in other studies [35,42,43,44]. In line with our results from the STZ rats, a lack of liver alterations has also been observed even 2 weeks after STZ induction [45]. On the other hand, the diabetogenic effect of STZ has been attributed to the selective and partial destruction of β cells, producing pancreatic damage [46]. The moderate number of insulin-positive cells in HFD + STZ pancreatic islets is in accordance with those reported in previous rat models with HFD + STZ [35,47]. In a similar vein, two studies involving the STZ model demonstrated that insulin-producing cells in the STZ model were relatively scarce, which is in accordance with our results [48,49].

By the analysis of these different metabolic, biochemical, and histological aspects, we confirmed that the use of HFD + STZ is a reliable model for the induction of T2D, as is STZ for T1D. The liver function plays a pivotal role by regulating de novo hepatic glucose production, among other metabolic processes. Therefore, we focused on screening the hepatic gene expression profile after T1D- and T2D-induction to provide a better understanding of the cellular and molecular pathways altered by chemical diabetes induction. A novel contribution of this study is the use of a robust, validated, and consistent platform with a precise Clariom D array rat (Affymetrix) to examine in parallel the gene expression profile of these T1D and T2D models.

According to the literature analysis, previous studies have evaluated the liver gene expression profile of animals with induced diabetes. However, we found, in these reports, different experimental conditions that could impact the stage and the severity of the disease and, consequently, modify the gene expression profile and its comparison. Additionally, another differential factor is the probe density of the microarray assay employed for the analysis. The differences found between this current study and a related study included a longer duration of HFD consumption after STZ administration (22 weeks, since these authors focused on the development of diabetic nephropathy) as well as the type of microarray assay, which only included 7407 mouse expression sequence tags [16]. In other related studies, the type of diet was different from ours (high-fat: 22% and high-carbohydrate: 48%, HFCHD), and the evaluation was performed with an expression BeadChip containing 22,226 oligonucleotides [50]. Furthermore, the gene expression profile of Otsuka Long-Evans Tokushima Fatty (OLETF) rats was reported; however, these results might not be comparable with ours, because OLETF rats belong to a spontaneous diabetes model [51]. Regarding the influence of STZ on the liver gene expression profile, our results are in line with Sadi et al. who reported a comparable number of DEG (273 genes), and their upregulation was related to several biological processes such as catalytic activities, oxidation–reduction reactions, co-enzyme binding, and terpenoid biosynthesis. On the other hand, downregulated genes were involved in carbohydrate metabolism, cell signal transduction, calcium-independent cell-to-cell adhesion, and lipid catabolism [15]. This study correlates well with our results, and this can be explained because the authors employed a similar analysis platform (Affymetrix rat genome 230 2.0 arrays platform), and 31,000 transcripts were able to be evaluated.

Moreover, our bioinformatic analysis, performed through STRING enrichment, indicate that upregulated genes in the STZ model (T1D) are associated with several aspects of lipid biosynthesis and metabolism. These findings align with the existing scientific literature. Emerging evidence underscores the significant role of abnormal lipid metabolism in the pathogenesis of T1D as well as in its progression [52]. Gene ontology of the biological processes in the hereto analyzed STZ induction includes genes related to cholesterol biosynthesis, sterol biosynthesis, and sterol and cholesterol metabolism. Accordingly, sterol lipids, including sterols, steroids, bile acids, and derivatives, were associated with T1D-related complications and T1D disease progression [52]. Similar to the data that we report above, previous studies have demonstrated changes in the liver metabolism of cholesterol, involving the reduced expression and secretion of apolipoproteins as well as a downregulation of LDL receptor gene expression [53,54]. These findings highlight the importance of lipids, particularly altered cholesterol metabolism, in STZ models. In addition, an effect related to targets involved in the function of peroxisomes was revealed. These single-membrane-bound organelles play an important role in cell metabolism through processes such as the alpha- and beta-oxidation of fatty acids, etherphospholipid biosynthesis, and glyoxylate detoxification [55]. Thus, peroxisomes regulate the accumulation and utilization of lipids. Altogether, these findings provide a closer insight into potential organelles involved in the disturbance of the lipid metabolism, as indicated by our microarray data.

On the other hand, using the STRING enrichment analysis, we found that the upregulated genes in the HFD + STZ model are associated with immunometabolism. This finding is supported by the existing scientific literature. Immunometabolism refers to the interplay between the immune system and metabolic processes, which can lead to the development and progression of obesity and diabetes. In obese patients and high-fat fed animal models, the presence of classical M1 macrophages in adipose tissue has been clearly linked to impaired insulin action. Beyond the innate immune system, it has been demonstrated that the adaptive immune response, involving T and B lymphocytes, can also influence metabolic processes. The crosstalk between the immune system and metabolism has been observed in various tissues, such as the liver, adipose tissue, gut, muscle, and pancreas, suggesting functional interactions with consequences for the development of diabetes. This multiorgan crosstalk involves various humoral factors and leads to several cellular changes depending on the tissue. In the liver, there is an M1 polarization of Kupffer cells, increased macrophage infiltration, increased neutrophil activity (neutrophil elastase), increased proinflammatory cytokine production, activation of PPAR-γ, NF-κB, TLR4 and inflammasomes. A comprehensive understanding of immunometabolism in T2D could provide new avenues for its therapeutic intervention [56].

Regarding the link between the phenotypes and the molecular signatures identified for each diabetes model, it is tempting to speculate on how the pathways identified by the bioinformatic analyses can explain the phenotypes observed in the STZ and HFD + STZ animals. An early observation consisted of a clear diverging effect of the STZ induction on the subsequent body weight of the animals. A potential target involved in the decrease in body weight in the STZ animals relates to the increased expression of the leptin gene found in our microarray data. The leptin hormone has been shown to influence insulin sensitivity and promote body weight loss [57]. Moreover, leptin exerts anti-steatotic effects by decreasing liver metabolic activity and increasing lipid exportation to other tissues [58]. This potential regulation of the leptin signaling also agrees with changes observed in the lipid profile of HFD + STZ animals, which exhibited an increase in leptin receptor expression and higher total cholesterol and LDL-cholesterol serum levels compared to those of control or STZ rats. Despite an augmentation in leptin receptor expression in HFD + STZ animals, this was not sufficient to prevent the insulin resistance observed in our results and this probably reflects a compensatory mechanism in the insulin–leptin axis [59].

Besides differences in weight gain, a distinct hyperglycemia stability was found in the models. This is likely related to the oxidative stress targets revealed by the liver microarray and the activation of liver detoxification systems which can exhibit interindividual variability and lead to the differential susceptibility often observed in STZ acute inductions [60,61]. In contrast, a chronic induction with the HFD-feeding seems to exert more consistent damage that results in a homogenous hyperglycemia probably as a consequence of the systemic insulin resistance (also corroborated by our experiments). Besides the classical proinflammatory pathway, TLR6 represents an interesting target identified in our experiments, which has been suggested to play a role in non-alcoholic fatty liver disease and hepatic inflammation [62].

In line with this, the liver histological analyses also supported a role for inflammatory processes that revealed lobular inflammation and prominent cell ballooning in HFD + STZ animals. On the other hand, the hepatic tissue from STZ animals displayed a normal histological liver architecture since the oxidative stress is likely to precede the development of a severe liver dysfunction. Regarding our transcriptomic findings and their association with the fatty liver phenotype, we observed an increase in *Plin2* gene expression in HFD + STZ animals, consistent with recent findings reported in the literature [63]. Notably, *Plin2* is a predominant member of the Plin protein family in both rat and human livers, and its expression correlates strongly with the severity of steatosis [64]. In contrast, our results from the STZ group revealed a decrease in *Plin2* expression, correlating with the absence of steatosis in livers from STZ rats.

Abnormal lipid metabolism has been shown to influence glucose homeostasis, as confirmed by our experiments showing both impaired glucose and insulin tolerance, particularly in HFD + STZ rats. *Insig1* and *Srebf1*, found downregulated and upregulated in our microarray for HFD + STZ animals, respectively, play a role in cholesterol biosynthesis. An overexpression of liver *Insig-1* reduces insulin-stimulated lipogenesis, inhibits SREBP signaling, and leads to decreased liver and plasma levels of cholesterol and triglycerides [65]. This agrees with our findings, that show an opposite pattern at the gene expression levels and increased circulating lipid concentrations in HFD + STZ rats. An interesting observation, related to glucose homeostasis, is the reduction in glucotransporter 4 (*Slc2a4*) gene expression in both STZ and HFD + STZ rats. This glucose transporter, although more widely described for adipose and muscle tissue, is relevant to the response in glucose uptake induced by a stimulus with insulin [66].

In our bioinformatics analysis of microarray data from both models, we also detected molecular targets associated with T1D and T2D in humans. Regarding our STZ animal model, we found some genes that exhibited a similar expression pattern in humans. For example, Pseudopodium Enriched Atypical Kinase 1 (*PEAK 1*) and S100 Calcium Binding Protein P (*S100P*) were among the top 10 upregulated genes in human T1D patients [67]. Accordingly, in that work, the authors also reported downregulated genes like proline Rich 5 Like (*PRR5L*), Growth Factor Augmenter of Liver Regeneration (*GFER*), CD 160 Molecule (*CD160*), Mitochondrial Ribosomal Protein S18C (*MRPS18C*), and Ubiquitin C-Terminal Hydrolase L3 (*UCHL3*) that we were also able to identify as under-expressed genes.

Likewise, our results coincided with some genes reported in human T2D patients. Among the top 10 upregulated genes, we corroborated these expression levels for the following genes: SOS Ras/Rac Guanine Nucleotide Exchange Factor 1 (*SOS1*), Early Growth Response 2 (*EGR2*), C-C Motif Chemokine Receptor 1 (*CCR1*), β-1,4-Galatosyltransferase 5 (*B4GALT5*), Ubiquitin Specific Peptidase 10 (*USP10*), Plexin C1 (*PLXNC1*), Purine Nucleoside Phosphorylase (*PNP*), and Exportin 7 (*XPO7*). With respect to the genes observed to be downregulated in our HFD + STZ model, the following genes were also correlated with human T2D: Adhesion Regulating Molecule 1 (*ADRM1*), Sorting Nexin 1 (*SNX1*), Insulin Receptor Substrate 1 (*IRS1*), DR1 Associated Protein 1 (*DRAP1*), and Uroporphyrinogen Decarboxylase (*UROD*) [67].

One limitation of this study is that we focused on exploring differential gene expression in the liver tissue of two experimental models of diabetes. Despite some phenotypic differences observed in the models being explainable by transcriptomic results, further studies are needed to corroborate these findings. Moreover, it would be interesting to further analyze the expression profile of pancreatic β-cells from both diabetes models in future studies. This will allow the identification of molecular changes occurring in pancreatic β-cells due to diabetes induction and compare them to data obtained in human islets. For example, a recent study reported the importance of RHOT1 for mitochondrial function and insulin secretion [68]. These findings will allow us to identify to a greater extent whether and how experimental models of diabetes resemble the human disease.

Finally, it has been reported that animals with induced diabetes exhibit a significant increase in oxidative stress and inflammatory markers. These findings are in agreement with our data. However, it would be worthwhile for future research to evaluate parameters related to oxidative stress, such as thiobarbituric acid reactive substances (TBARS), glutathione (GSH), superoxide dismutase (SOD), catalase (CAT), and advanced glycation end-products (AGEs). Moreover, the assessment of inflammatory biomarkers, such as tumor necrosis factor alpha (TNF-α), interleukin-6 (IL-6), interleukin-1β (IL-1β), and nuclear factor Kappa B (NF-κB), could also provide valuable insights [69].

## 4. Materials and Methods

### 4.1. Animals

**Figure 7:**
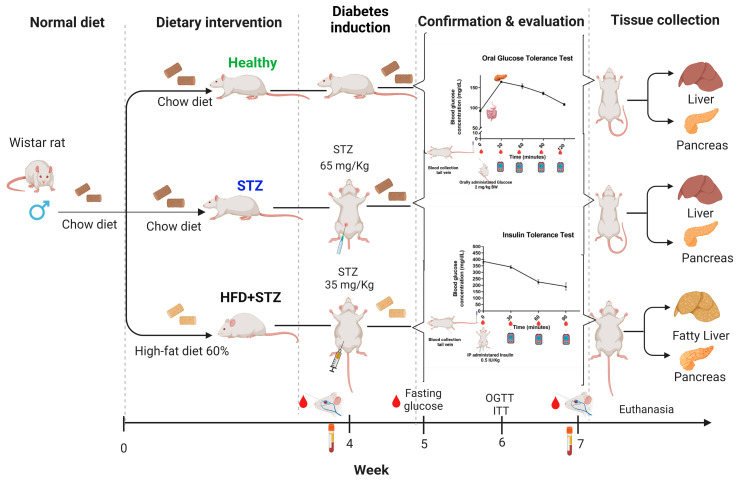
Experimental design and timeline of the diabetes induction scheme. Rats were allocated to three experimental groups, with 9 animals per group: the healthy group (animals without induction, fed with a standard chow diet), the STZ group (animals fed with a standard chow diet that received a single i.p. STZ dose (65 mg/kg BW)), and the HFD + STZ group (animals fed with an HFD for 4 weeks before a single i.p. administration of STZ at a low dose (35 mg/kg BW)). Once hyperglycemia was corroborated (fifth week), we performed an evaluation of the glucose homeostasis with insulin and oral glucose tolerance tests (ITT and OGTT, respectively) between the fifth and seventh week, with a difference of 7 days in the performance of ITT and OGTT. Abbreviations: STZ, streptozotocin; HFD, high-fat diet; i.p., intraperitoneal; BW, body weight.

A total of 27 male Wistar rats between 6 and 8 weeks of age (150–220 g of body weight, BW) were obtained from the Universidad de Guadalajara bioterium. The animals were maintained under conditions of 24 ± 2 °C, 55.5 ± 5% humidity, with light and dark cycles of 12 h each, and provided ad libitum access to water and a standard diet. After acclimation for 1 week, the rats were randomly assigned into three experimental groups: the healthy control group, without induction (n = 9), the STZ group (n = 9), and the HFD + STZ group (n = 9) (Figure 7). The experimental protocol was reviewed and approved (registration number CI-01919) by the Universidad de Guadalajara (Research, Bioethics, and Biosecurity Committees), and the procedures were conducted according to the Guidelines for Care and Use of Laboratory Animals of the Mexican Federal Regulation (NOM-062- ZOO-1999, updated in 2001).

### 4.2. Experimental Diabetes Induction

#### 4.2.1. Type 1 Diabetes Mellitus Induction (STZ Model)

Diabetes was induced with a single intraperitoneal (i.p.) injection of STZ (65 mg/kg BW) (cat. S0130-1G, Sigma-Aldrich, St. Louis, MO, USA) dissolved in 0.1 M sodium acetate buffer, pH 4.5 [6,70].

#### 4.2.2. Type 2 Diabetes Mellitus Induction (HFD + STZ Model)

Diabetes was induced by feeding animals an HFD [9] for 4 weeks. Then, the animals were fasted for 12 h and given intraperitoneally a unique dose of streptozotocin (STZ, 35 mg/kg BW) (cat. S0130-1G, Sigma-Aldrich, St. Louis, MO, USA), freshly diluted in a 0.1 M sodium citrate buffer, pH 4.5.

#### 4.2.3. Formulation of the High-Fat Diet (HFD)

A total of 100 g of high-fat diet, prepared by ourselves, contained 23.0 g of casein (cat. 400601, Dyets, Inc., Bethlehem, PA, USA), 2.4 g of vitamin mix (AIN-93VX cat. 310025, Dyets, Inc., Bethlehem, PA, USA), 8.0 g of a mineral mix (AIN-93G-MX Mineral Mix, cat. 210025, Dyets, Inc., Bethlehem, PA, USA), 33.4 g of cholesterol (cat. 400650, Dyets, Inc., Bethlehem, PA, USA), 18.0 g of cornstarch, 10.1 g of sucrose, 34.0 g of lard, and 1.1 g of crude sodium cholate (cat. S9875-500G, Sigma-Aldrich, St. Louis, MO, USA). The lard consisted mainly of saturated (37%) and monounsaturated (45%) fatty acids. In addition, a proportion of cholesterol and sodium cholate was added. In contrast, the standard chow (rodent Lab Chow diet 5001, Labdiet, St. Louis, MO, USA) diet provided 3.3 kcal per gram (13.4% kcal from fat) [9,71].

#### 4.2.4. Diabetes Confirmation after Chemical Induction

Diabetes was confirmed in both models by measuring the fasting glucose concentration (glucose ≥200 mg/dL) 1 week after STZ administration.

### 4.3. Oral Glucose Tolerance Test (OGTT)

Healthy animals were subjected to a 12 h fasting period and, subsequently, they received a glucose solution (2 g/kg BW) through an oral feeding cannula. Before exogenous glucose administration (time 0), a tail puncture was performed for blood glucose quantification with a glucometer (Accuchek, Roche, Germany). Thereafter, blood glucose measurements were performed at 30, 60, 90, and 120 min.

The glycemia follow-up was performed at the end of the experiment in all groups. Animals that were induced to diabetes were not subjected to a 12 h fast period, since, at the beginning, we observed an instability in the glucose values exhibited by STZ animals in a period of fasting, reaching even hypoglycemic values. In addition, we did not expose the diabetic animals to a glucose challenge to ensure detectable glucose values by the glucometer (under 599 mg/dL) during the assay, since these animals exhibited fasting blood glucose levels higher than 400 mg/dL.

With the glycemia levels from the curve, we calculated the area under the curve (AUC) values using the trapezoidal rule.

### 4.4. Insulin Tolerance Test (ITT)

The animals were subjected to a 12 h fasting period and injected with a single i.p. dose of 0.5 IU/kg BW human recombinant insulin (PiSA Farmaceutica, Mexico, Mexico). Blood glucose concentration was quantified at 0, 30, 60, 90, and 120 min with a glucometer.

### 4.5. Blood and Tissue Collection

The animals were fasted for 12 h and anesthetized with isoflurane. Peripheral blood samples were collected from the retro-orbital plexus. Serum was separated by centrifuging the blood samples (3500 rpm, 15 min, 4 °C) and stored in aliquots at −80 °C until analysis. The liver and pancreas, from animals of each group, were collected for further analysis.

### 4.6. Biochemical Parameters

The serum levels of the biochemical parameters were determined by colorimetric assays using biochemical reagents from Biosystems (Barcelona, Spain) and a semi-automated spectrophotometer (BTS-350; BioSystems, Spain). Serum levels of glucose (Cat. 11504), alanine aminotransferase (ALT, Cat. 11533), aspartate aminotransferase (AST, Cat. 11531), urea (Cat. 11537), creatinine (Cat. 11802), cholesterol (Cat. 11506), high-density lipoprotein cholesterol (HDL-c, Cat. 11648), low-density lipoprotein cholesterol (LDL-c, Cat. 11579), and triglycerides (Cat. 11529) were quantified before (pre) and after (post) diabetes induction. In the case of healthy animals, we analyzed these parameters at the beginning and after 3 weeks of the experiment.

### 4.7. Liver Index

The liver organs were individually weighed immediately after their extraction. Subsequently, we calculated the liver index to determine the ratio of the liver weight to the body weight of the animal, as an objective parameter of comparison. Liver index values were expressed as a percentage (%).

### 4.8. Histopathology Assessment

After euthanasia, the liver and pancreas were extracted and fixed in 4% of paraformaldehyde in 1X phosphate-buffered saline (PBS) and embedded in paraffin blocks. Histological sections of 5 µm were obtained with a microtome (Leica^®^, series 050131379, cat. 14050237993) and stained either with hematoxylin–eosin (H&E) or Masson’s trichrome. A certified pathologist performed the histopathological analysis.

### 4.9. Insulin Immunohistochemistry (IHC)

To determine the insulin content in pancreatic β cells, paraffin-embedded tissues were cut (4 µm) and these sections were incubated overnight at 4 °C with a 1:1000 dilution of rabbit monoclonal antibody against rat insulin (Cell Signaling Technology, Danvers, MA, USA). Detection was performed with a secondary antibody provided in the Mouse/Rabbit ImmunoDetector HRP/DAB Detection System (BIO SB, Hercules, CA, USA). Subsequently, the sections were counterstained with H&E. For negative controls, the primary antibodies were replaced with 1X PBS. The images of the pancreas slides immunostained with the antibody against insulin were analyzed using ZEN microscopy software (version 3.2, Carl Zeiss, Jena, Germany). A pathologist skilled in pancreatic morphometric analysis performed the semiquantitative interpretation, reading through the whole sample in the slide and assigning percentages to each staining degree and intensity of insulin-positive cells in the Langerhans islets. The identities of the samples from the control and the experimental groups were kept unknown to the pathologist during this analysis.

### 4.10. RNA Extraction

An RNeasy^®^ Mini Kit Cat. 74,106 (Qiagen, Hilden, Germany) was used to extract total RNA from liver tissue following the manufacturer’s recommendations. Spectrophotometry was used to determine the concentration and purity of RNA (Nanodrop 2000 spectrophotometer (Thermo Fisher Scientific, Waltham, MA, USA). Using denaturing agarose gel electrophoresis and microfluidic analysis with an Agilent 2100 Bioanalyzer (Agilent Technologies, Santa Clara, CA, USA) and Agilent RNA 6000 Nano Kit Part Number 5067-1511 (Agilent Technologies, Santa Clara, CA, USA), the RNA integrity of the samples was assessed.

### 4.11. DNA Microarray

For each experimental group (healthy, HFD + STZ, and STZ groups), we prepared three biological replicates of total RNA pools from the liver. Each pool consisted of RNA isolated from three different animals. The samples were processed and hybridized to Affymetrix’s Clariom^TM^ D Rat microarray chips (Affymetrix, Santa Clara, CA, USA) in the Microarray Unit of the National Institute of Genomic Medicine (INMEGEN) in Mexico. The hybridization and scanning of the microarray were performed according to GeneChip^TM^ Whole Transcript (WT) Expression Arrays User Guide of Affymetrix Corporation. Clariom D Rat is a photolithographic microarray platform consisting of printed oligonucleotide sequences on a glass-substrate formatted cartridge. Initially, total RNA was primed using primers containing a T7 promoter sequence at the 5′ end to synthesize a first-strand cDNA by reverse transcription. Next, a second-strand cDNA was synthesized with a DNA polymerase and the RNA was degraded by the RNAase H. The synthesis of the second-strand cDNA results in a double-stranded cDNA that is used as a template for the in vitro transcription of an antisense or complementary RNA (cRNA, RT-IVT method). The cRNA was purified, quantified, and employed for a second cycle of single-stranded cDNA (ss-cDNA) synthesis by reverse transcription including dUTP in the reaction mixture. RNase H was added to remove cRNA. The ss-cDNA was purified and fragmented using Uracil-DNA Glycosylase (UDG), and Apurinic/Apyrimidinic Endonuclease 1 (APE1) at the unnatural dUTP residues. The fragmented cDNA was labeled with Terminal Deoxynucleotidyl Transferase (TdT) and biotinylated labeling reagents. Then, 5.2 µg of fragmented and biotinylated ss-cDNA were added to the hybridization master mix. The hybridization cocktails (containing the ss-cDNA and the hybridization master mix) were prepared and heated at 9 9 °C for 5 min followed by 5 min at 45 °C before injecting 200 µL into the Clariom D Rat array. The arrays were incubated in a GeneChip Hybridization Oven 645, rotating at 60 rpm for 16 h at 45 °C. After hybridization, the arrays were stained and washed using a GeneChip Hybridization, Wash, and Stain Kit (Thermo Fisher Scientific REF 900720) and a GeneChip Fluidics Station 450. Finally, the arrays were scanned using a GeneChip Scanner 3000 7G System (Affymetrix, Santa Clara, CA, USA) according to the manufacturer’s instructions (Affymetrix, Santa Clara, CA, USA). CEL and CHP data files were generated and processed with the Affymetrix software (Transcriptome Analysis Console, TAC, v.4.0.2, ThermoFisher Scientific, Santa Clara, CA, USA) for further bioinformatic analysis.

### 4.12. Bioinformatic Analysis

The gene expression files were processed and analyzed with the Applied BioSystems Transcriptome Analysis Console 4.0.2 (TAC, ThermoFisher, Santa Clara, CA, USA). TAC Software is designed to develop meaningful insights from transcriptomic microarray (array) data using a variety of statistical, visualization, and quality control (QC) tools. To normalize the gene expression data, and allow for differential gene expression analysis, predetermined parameters were employed: Robust Multichip Average (RMA), Detected Above Background (DABG), *Rattus norvegicus,* and eBayes as the ANOVA method. In addition, we selected the fold change value < −2 or > 2, (*p*-value < 0.05), as a filter setting for data analysis. To assess the distribution of the triplicates from all groups, principal component analysis (PCA) and hierarchical clustering, provided by TAC, were used. In the transcriptome analysis, the group sample signals of each gene were compared to all groups to identify which coding genes modify their expression in comparison to the healthy group as reported before [72], and how some genes are expressed oppositely between both diabetic groups. For functional enrichment analysis, STRING database (version 11.5) was used to analyze differentially expressed target interactions. Databases such as Gene Ontology (GO), Mammalian Phenotype Ontology (Monarch), the Kyoto Encyclopedia of Genes and Genomes (KEGG), and WikiPathways were used to perform the analysis to identify the cell localization, main cellular pathways and molecular networks influenced by the induction of diabetes.

### 4.13. Statistical Analysis

The data were expressed as the mean ± standard error of the mean (SEM). The intra-group differences in the biochemical parameters pre- and post- induction were analyzed with a paired *t*-test. A *p* value <0.05 was considered significant. The results from the area under the curve (AUC) were analyzed by one-way analysis of variance (ANOVA) followed by the Tukey post hoc test. The graphing and calculation of the AUC analysis were performed using GraphPad Prism Software (version 8.0.1) (San Diego, CA, USA).

## 5. Conclusions

In this study, we found that the HFD + STZ model exhibits a stable-over time phenotype to maintain a hyperglycemic range compared to the STZ model. Insulin resistance, a key risk factor for NAFLD, obesity, and T2D, was observed in HFD + STZ animals. Our data confirmed the phenotypic alterations observed in STZ as a T1D model and HFD + STZ as a T2D model. Moreover, using a robust, validated, and consistent microarray platform, we examined the liver gene expression profile and identified coding genes with contrasting expression patterns between the experimental STZ and HFD + STZ diabetes induction groups. Subsequent enrichment analyses of these genes revealed pathways related to lipid biosynthesis and metabolism in the STZ model, whereas immunometabolism pathways were associated to the HFD + STZ model. Despite some phenotypic differences observed in the models being explainable by transcriptomic results, further studies are needed to corroborate these findings. We also provide genetic data from healthy rats and these two experimental diabetes models: STZ and HFD + STZ, which could be an approach as a reference in future experimental studies for a better understanding of the pathology and to test novel treatments of diabetes. Further studies assessing oxidative stress as well as inflammatory pathways are necessary to dilucidate key molecules promoting differential gene expression and phenotypes in STZ and HFD + STZ models.

## Figures and Tables

**Table 1:** Biochemical parameters pre- and post-induction in the experimental groups.

	Experimental Groups					
	Healthy Animals		HFD + STZ Model		STZ Model	
	Initial Time	Final Time	Pre- Induction	Post- Induction	Pre- Induction	Post- Induction
Glucose (mg/dL)	125.0 ± 6.4	116.8 ± 8.8	169.6 ± 10.2	282.4 ± 46.6 *	141.4 ± 5.2	215.6 ± 34.1 *
Triglycerides (mg/dL)	53.0 ± 6.6	46.4 ± 4.7	77.6 ± 4.5	156.2 ± 33.9	67.6 ± 1.8	91.4 ± 6.5 *
Cholesterol (mg/dL)	59.8 ± 10.5	57.8 ± 8.8	78.0 ± 3.4	171.3 ± 18.4 *	50.0 ± 8.6	59.4 ± 10.5
HDL-c (mg/dL)	43.4 ± 10.4	42.3 ± 2.0	39.0 ± 2.8	41.8 ± 3.7	44.2 ± 1.6	49.5 ± 8.5
LDL-c (mg/dL)	5.8 ± 0.8	12.2 ± 7.8	18.9 ± 3.4	119.3 ± 16.9	15.4 ± 9.5	13.0 ± 7.3
AST (U/L)	58.7 ± 7.6	54.7 ± 2.7	63.0 ± 6.4	46.0 ± 8.9	58.7 ± 3.6	38.5 ± 4.7
ALT (U/L)	34.5 ± 3.2	29.6 ± 2.1	69.8 ± 17.1	75.3 ± 25.1	55.3 ± 15.5	29.0 ± 2.8
Urea (mg/dL)	46.6 ± 6.6	42.4 ± 2.0	55.6 ± 7.8	41.0 ± 5.2	58.0 ± 5.5	75.6 ± 4.8 *
Creatinine (mg/dL)	0.6 ± 0.0	0.6 ± 0.0	0.6 ± 0.1	0.9 ± 0.1 *	0.7 ± 0.1	0.6 ± 0.0

HDL-c, high-density lipoprotein cholesterol; LDL-c, low-density lipoprotein cholesterol; AST, aspartate aminotransferase; ALT, alanine aminotransferase. HFD + STZ: high-fat diet plus streptozotocin, STZ: streptozotocin. Values represent mean ± standard error of the mean (SEM), n = 9 animals per experimental group. * *p* < 0.05.

**Table 2:** Top ten differentially regulated coding genes.

Healthy vs. HFD + STZ		Healthy vs. STZ		HFD + STZ vs. STZ	
Upregulated					
Gene	Fold Change	Gene	Fold Change	Gene	Fold Change
	(log2)		(log2)		(log2)
*Idi1*	32.44	*Stac3*	10.89	*Mmp12*	36.32
*Msmo1*	19.65	*Omd*	3.19	*Clec7a*	25.64
*Cyp51*	17.87	*Car3*	2.88	*Scd1*	25
*Inmt*	17.07	*Omd*	2.87	*Lilrb4*	23.21
*Sqle*	15.97	*Cdh17*	2.87	*Gpnmb*	21.67
*Tm7sf2*	14.35	*Tstd1*	2.84	*Lpl*	21.35
*Stac3*	12.03	*Scd1*	2.67	*Spp1*	16.45
*Hmgcs1*	9.42	*Rps14*	2.57	*Pla2g7*	16.13
*Acss2*	7.7	*Fabp7*	2.38	*Scd2*	10.84
*Hsd17b7*	6.49	*Rps17l*	2.36	*Wfdc21*	10.33
Downregulated					
Gene	Fold Change	Gene	Fold Change	Gene	Fold Change
	(log2)		(log2)		(log2)
*Abcb1b*	−33.97	*Abcc3*	−7.98	*Idi1*	−30.24
*Gpnmb*	−32.99	*Cyp1a1*	−7.9	*Msmo1*	−29.6
*Mmp12*	−27.99	*Cyp17a1*	−6.72	*Sqle*	−23.97
*Lpl*	−23.83	*Acmsd*	−6.71	*Cyp51*	−21.37
*Clec7a*	−20.92	*Elovl6*	−5.68	*Elovl6*	−12.76
*Lilrb4*	−18.13	*Cyp2c12*	−5.3	*Hmgcs1*	−12.37
*Pla2g7*	−17.45	*Ppif*	−4.87	*Tm7sf2*	−11.68
*Spp1*	−13.96	*Elovl6*	−4.47	*Hsd17b7*	−11.4
*Scd2*	−13.38	*Ppm1l*	−4.39	*Prlr*	−10.25
*Lcn2*	−11.99	*Prlr*	−4.39	*Inmt*	−9.49

**Table 3:** Enrichment analysis of upregulated genes in the STZ model.

Gene Ontology (GO) Analysis			
Term	Description	Strength (log 10)	False Discovery Rate (*p* Value)
Biological processes			
GO:0010312	Detoxification of zinc ion	1.69	0.0452
GO:0006695	Cholesterol biosynthetic process	1.15	6.31 × 10^−7^
GO:0016126	Sterol biosynthetic process	1.12	2.26 × 10^−7^
GO:0016125	Sterol metabolic process	0.96	8.27 × 10^−12^
GO:0008203	Cholesterol metabolic process	0.95	1.12 × 10^−10^
Molecular function			
GO:0098809	Nitrite reductase activity	1.45	0.0454
GO:0032934	Sterol binding	0.87	0.0099
GO:0050660	Flavin adenine dinucleotide binding	0.71	0.0454
GO:0016491	Oxidoreductase activity	0.43	3.16 × 10^−5^
GO:0016740	Transferase activity	0.24	0.0013
Cellular components			
GO:0005778	Peroxisomal membrane	0.84	0.0050
GO:0005777	Peroxisome	0.56	0.0287
GO:0031968	Organelle outer membrane	0.48	0.0272
GO:0031301	Integral component of organelle membrane	0.44	0.0048
GO:0019866	Organelle inner membrane	0.38	0.0025
Mammalian Phenotype Ontology (Monarch)			
MP:0010161	Decreased brain cholesterol level	1.45	0.0167
MP:0010026	Decreased liver cholesterol level	1.08	0.00085
MP:0003983	Decreased cholesterol level	0.92	0.0020
MP:0012776	Abnormal liver cholesterol level	0.88	0.0014
MP:0003119	Abnormal digestive system development	0.74	0.0240
KEGG pathways			
mmu00100	Steroid biosynthesis	1.39	6.36 × 10^−8^
mmu00920	Sulfur metabolism	1.25	0.0119
mmu00900	Terpenoid backbone biosynthesis	1.03	0.0119
mmu04950	Maturity onset diabetes of the young	0.96	0.0190
mmu04979	Cholesterol metabolism	0.85	0.0119
WikiPathways			
WP103	Cholesterol biosynthesis	1.51	2.27 × 10^−9^
WP4346	Cholesterol metabolism with Bloch and Kandutsch−Russell pathways	1.18	5.53 × 10^−11^
WP1251	Metapathway biotransformation	0.66	0.00085
WP447	Adipogenesis genes	0.57	0.0289
Reactome Pathways			
MMU-6807062	Cholesterol biosynthesis via lathosterol	1.57	0.0486
MMU-6807047	Cholesterol biosynthesis via desmosterol	1.57	0.0486
MMU-191273	Cholesterol biosynthesis	1.41	2.37 × 10^−11^
MMU-9603798	Class I peroxisomal membrane protein import	1.09	0.0249
MMU-8957322	Metabolism of steroids	0.9	6.59 × 10^−9^

**Table 4:** Enrichment analysis of upregulated genes in the HFD + STZ model.

Gene Ontology (GO) Analysis			
Term	Description	Strength (log 10)	False Discovery Rate (*p* Value)
Biological Processes			
GO:0097527	Necroptotic signaling pathway	1.49	0.0258
GO:0006002	Fructose 6-phosphate metabolic process	1.42	0.0057
GO:0002467	Germinal center formation	1.4	0.0360
GO:0002220	Innate immune response activating cell surface receptor signaling pathway	1.39	0.0014
GO:0018158	Protein oxidation	1.36	0.0420
Molecular Function			
GO:0030246	Carbohydrate binding	0.75	3.93 × 10^−5^
Cellular Components			
GO:0009897	External side of plasma membrane	0.63	4.67 × 10^−6^
GO:0098552	Side of membrane	0.53	3.65 × 10^−5^
GO:0009986	Cell surface	0.51	1.40 × 10^−6^
GO:0031984	Organelle subcompartment	0.35	0.0050
GO:0005783	Endoplasmic reticulum	0.28	0.0115
Mammalian Phenotype Ontology (Monarch)			
MP:0011076	Increased macrophage nitric oxide production	1.79	0.0309
MP:0008473	Abnormal spleen follicular dendritic cell network	1.74	0.0032
MP:0020309	Increased creatine kinase activity	1.67	0.0425
MP:0012448	Abnormal primary motor cortex morphology	1.67	0.0425
MP:0008615	Decreased circulating interleukin-17 level	1.67	0.0425
KEGG Pathways			
mmu00524	Neomycin, kanamycin and gentamicin biosynthesis	1.74	0.0036
mmu00052	Galactose metabolism	1.06	0.0127
mmu00520	Amino sugar and nucleotide sugar metabolism	0.98	0.0089
mmu05321	Inflammatory bowel disease	0.97	0.0036
mmu04672	Intestinal immune network for IgA production	0.96	0.0248
WikiPathways			
WP4474	Circulating monocytes and cardiac macrophages in diastolic dysfunction	1.67	0.0472
WP113	TGF-beta signaling pathway	1.17	3.65 × 10^−5^
WP1253	Type II interferon signaling (IFNG)	1.08	0.0256
WP3625	Tyrobp causal network in microglia	0.98	0.0042
WP2432	Spinal cord injury	0.93	0.00023
Reactome Pathways			
MMU-5621480	Dectin-2 family	1.54	0.0448
MMU-5668541	TNFR2 non-canonical NF-kB pathway	0.93	0.0011
MMU-1280215	Cytokine Signaling in Immune system	0.6	0.0011
MMU-168249	Innate Immune System	0.56	1.98 × 10^−8^
MMU-6798695	Neutrophil degranulation	0.55	0.0011

## Data Availability

The data discussed in this publication have been deposited in NCBI’s Gene Expression Omnibus [73] and are accessible through GEO Series accession number GSE216668 (https://www.ncbi.nlm.nih.gov/geo/query/acc.cgi?acc=GSE216668, accessed on 1 November 2023).

## Supplementary Materials

The following supporting information can be downloaded at: https://www.mdpi.com/article/10.3390/ijms25084151/s1.
