# Supplementary material for: Comparative Screening of the Liver Gene Expression Profiles from Type 1 and Type 2 Diabetes Rat Models
Source: Int J Mol Sci. 2024 Apr 9;25(8):4151. doi: 10.3390/ijms25084151 (PMC11050131; doi:10.3390/ijms25084151)
Supplement: Supplementary file 1 [file ijms-25-04151-s001.zip › Supplementary_material_ijms-2912760.docx]

**Comparative screening of the liver gene expression profiles from type 1 and type 2 diabetes rat models**

Guerra-Ávila *et al.*

RNA Integrity Number (RIN) of liver RNA samples from experimental groups.

The integrity of the total RNA isolated from liver tissue of the three animal groups (healthy, HFD+STZ, and STZ) was assessed using a 2100 Bioanalyzer (Agilent, USA). Further processing with the Clariom D rat microarray chips requires a minimum RNA Integrity Number (RIN) of 8.0 (considered as acceptable). The samples of these experiments achieved a RIN ranging from 8.8 to 9.9. The quality of the RNA, as determined by digital gel electrophoresis is depicted in Figure S1 and Figure S2 for the HFD+STZ and STZ samples, respectively.

*
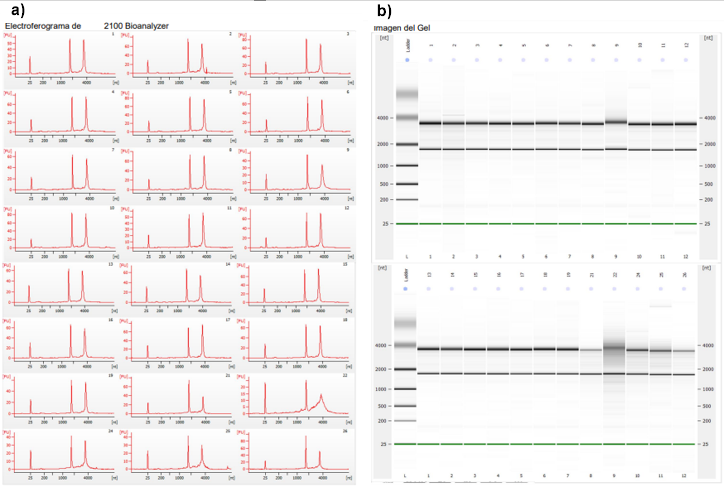
*

**Figure S 1.** Representative evaluations of RNA quality and integrity of samples from the healthy, and HFD+STZ groups are shown. In panel a), we show data obtained from the electropherograms and in panel b) we include the results from the digital gel electrophoresis.


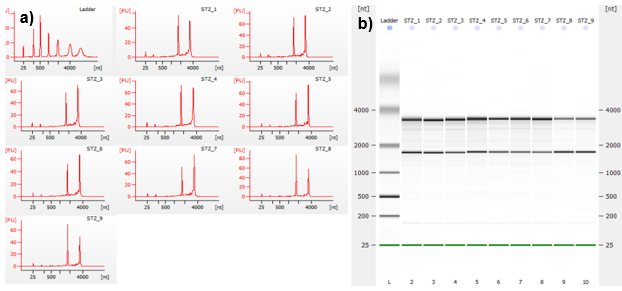


**Figure S 2.** Representative evaluations of RNA quality and integrity of samples from the STZ groups are shown. In panel a), we show data obtained from the electropherograms and in panel b) we include the results from the digital gel electrophoresis.

## Diabetes-related genes exhibiting opposed expression levels in the induced groups.

We compared the group sample signals (expression level) from each gene using the transcriptome analysis console (TAC). The clusters of genes exhibiting opposing expression levels in STZ and STZ+HFD are summarized in Figure S3 and S4, respectively.


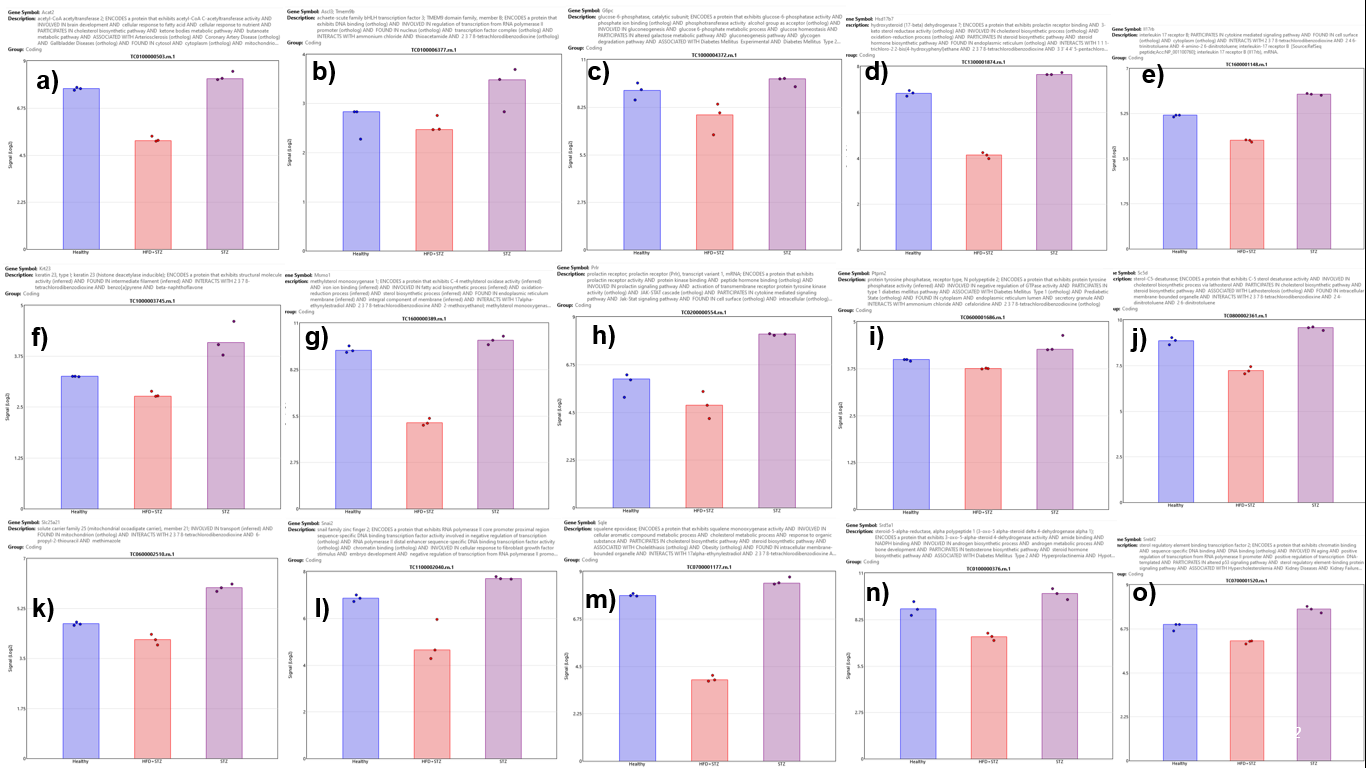


**Figure S 3**. Illustrative examples of cluster of 849 coding genes that exhibit opposite expression. Sample signals of upregulated genes from the STZ model were determined by the Transcriptional Analysis Console (TAC) from Affymetrix software. Bars show Healthy untreated group (blue), HFD+STZ group (pink) and, STZ group (purple bars), **a)** Acat2, **b)** Ascl3, **c)** G6pc, **d)** Hsd17b7, **e)** II17rb, **f)** Krt23, **g)** Msmo1, **h)** Prlr, **i)** Ptprn2, **j)** Sc5d, **k)** Slc25a21, **l)** Snai2, **m)** Sqle, **n)** Srd5a1, **o)** Srebf2.


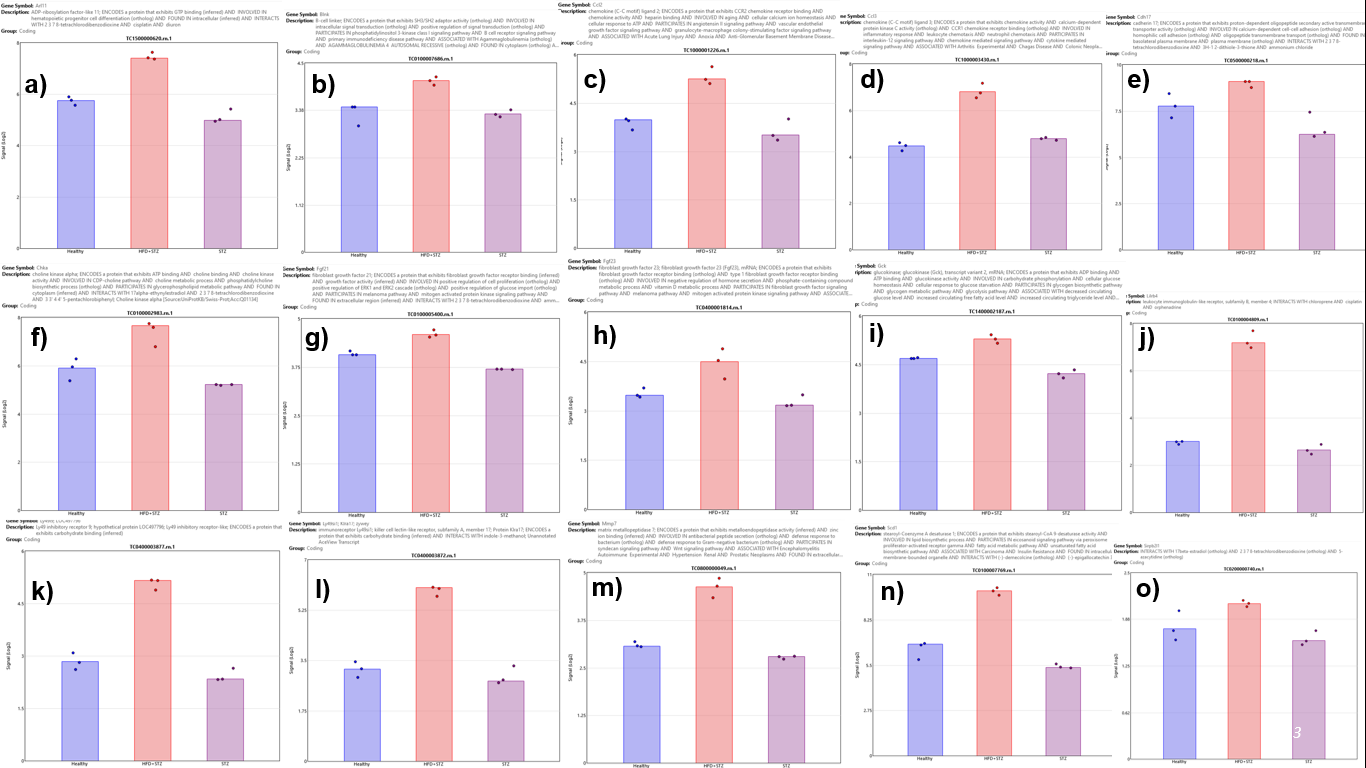


**Figure S 4.** Examples of cluster of 849 coding genes that exhibit opposite expression. Sample signals of upregulated genes from the HFD+STZ model were identified by the Transcriptional Analysis Console (TAC) from Affymetrix. Bars show Healthy untreated group (blue), HFD+STZ group (pink) and, STZ group (purple bars). **a)** *Arl11,* **b)** *Blnk,* **c)** *Ccl2,* **d)** *Ccr1***e),** *Cdh17,* **f)** *Chka,* **g)** *Fgf21,* **h)** *Fgf23,* **i)** *Gck,* **j)** *Lilrb4,* **k)** *Ly49i9,* **l)** *Ly49si1,* **m)** *Mmp7,* **n)** *Scd1,* **o)** *Sirpb2l1.*
